# Editorial: Celebrating the 200th mendel’s anniversary: gene-targeted diagnostics and therapies for cancer

**DOI:** 10.3389/fmmed.2024.1366963

**Published:** 2024-02-27

**Authors:** Noah Federman, Erlinda M. Gordon, Sant P. Chawla, Frederick L. Hall

**Affiliations:** ^1^ David Geffen School of Medicine, University of California, Los Angeles, Los Angeles, CA, United States; ^2^ Cancer Center of Southern California, Santa Monica, CA, United States; ^3^ Aveni Foundation, Santa Monica, CA, United States; ^4^ Counterpoint Biomedica LLC, Santa Monica, CA, United States; ^5^ Delta Next-Gene, LLC, Santa Monica, CA, United States

**Keywords:** Cyclin G1, DeltaRex-G, sarcoma, pancreatic cancer, carcinoma of breast, whole genome sequencing, liquid biopsy, gene therapy

**FIGURE 1:**
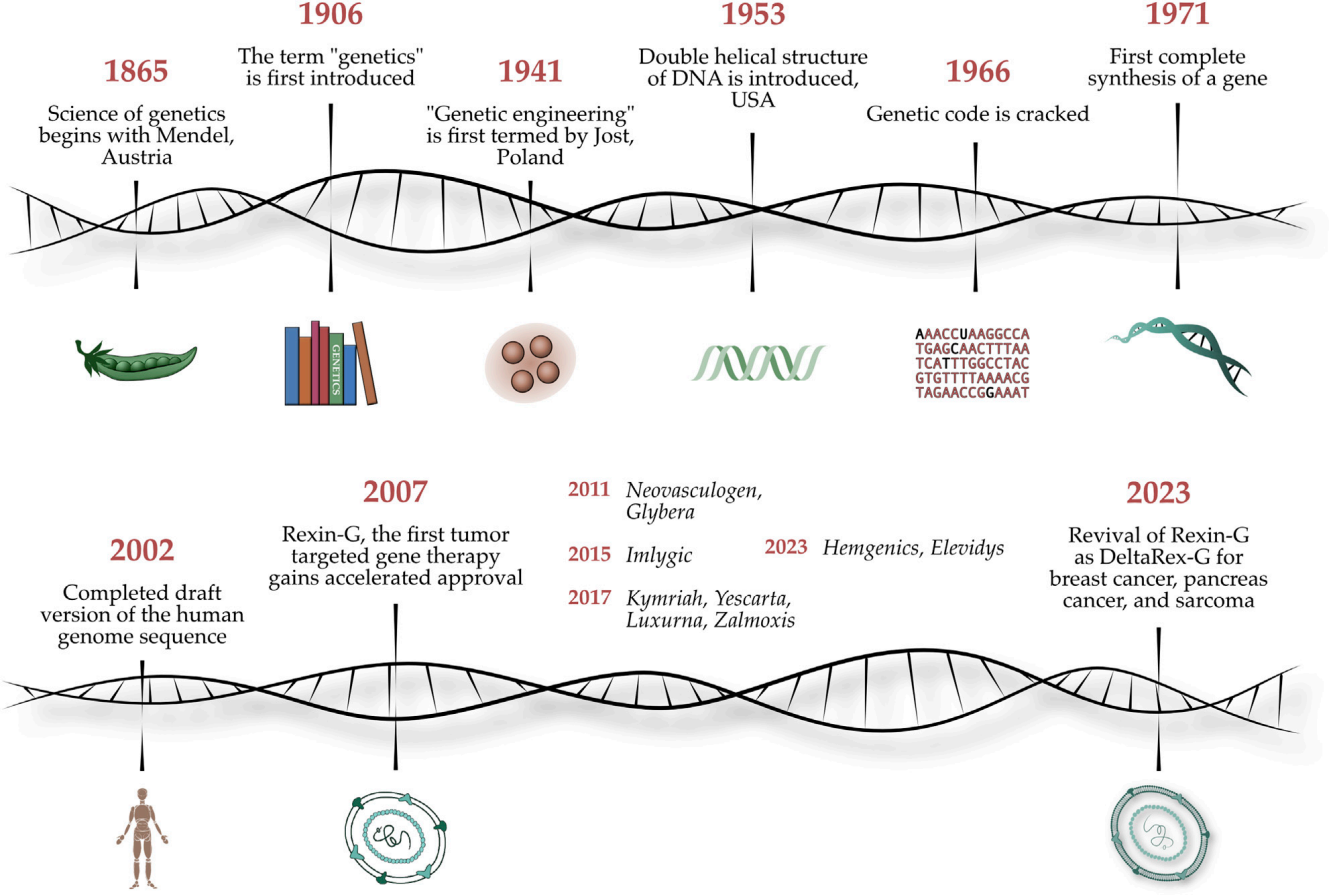
Legend: Celebrating Mendel’s 200th Anniversary. An artist’s illustration of the chronology of events from Gregor Mendel’s experiments on pea plants to the discovery of the genetic code to whole genome sequencing, to genetic engineering of viral vectors for gene therapy applications (Jeffrey et al. 2023).

In a bicentennial celebration of the quantitative analytical investigations of Gregor Mendel (1822–1884)—which established the basic principles of heredity and the scientific foundations of classical genetics—we present a Special Research Topic entitled *Gene-Targeted Diagnostics and Therapies for Cancer*, which highlights the latest translations of molecular genetics, functional genomics, and applied proteomics into cancer therapy at the cutting-edge of precision medicine. Figure 1 shows an artist’s illustration of the chronology of events from Gregor Mendel’s experiments on pea plants to the discovery of the genetic code to genetic engineering of viral vectors for gene therapy applications (Jeffrey et al. 2023).

Over the years, the concepts of classical genetics have necessarily been enlarged in scope to include the emergent concepts of chromosome pairs, the DNA double helix, the genetic code, epigenetics, the discovery of split-genes (introns and exons), the vast preponderance of non-coding/regulatory RNA/transcribed sequences within the human genome (Gayon, 2016), and the myriad of executive biochemical pathways (Yasutis and Kozminski, 2013) that regulate mammalian gene expression. Indeed, the availability of advanced DNA sequencing technologies has served to identify the functional gene regulatory elements (including promoters, enhancers, silencers, and insulators) which, along with the cognate transcription factors, regulate and coordinate gene transcription; and yet, when mutated or otherwise dysregulated, can become major drivers of human disease (Chatterjee and Ahituv, 2017).

In time, the identification and characterization of cellular proto-oncogenes, the concept of oncogene addictions, and the loss of tumor suppressors observed in the processes of multistep carcinogenesis—that is, in the *initiation*, *promotion*, and *progression* of metastatic cancers—came to be viewed cytogenetically as *heritable traits* in the pathogenesis and evolution of solid tumors, seen as the evolving populations of metastatic cancer cells (Al-Shihabi et al., 2018; Grzes et al., 2020). The topical paper presented by Zou et al. demonstrates the power of Whole Genome Sequencing (WGS) and patient-matched samples in a rare, highly recurrent soft tissue sarcoma to examine genomic alterations, clonal evolution, and mutation rates in primary tumors, when compared with metastatic and recurrent tumor samples, to gain insights into the molecular pathogenesis, disease staging, and potential therapeutic strategies.

Among the Research Topic of topical papers presented herein is a timely retrospective review from Gordon and Hall who previously identified the executive components of a functional “proline-directed” protein kinase-mediated signal transduction cascade—from the human EGFR-associated/p38 Mapk14 kinase (Williams et al., 1993), to the human Cdk-activating kinase (Wu et al., 1994a), to the human Cdk2 (CDK2) kinase (Elledge et al., 1992), to the human Cyclin G1 (CYCG1) oncogene (Wu et al., 1994b), to the Cyclin G1-binding p18 (FX3) gene product, aka p18-Hamlet (Xu et al., 2000)—characterizing the Cyclin G1(CCNG1) oncogene as a pivotal and commanding locus: revealing the *Cyclin G1 Axis* of protein-protein interactions (PPIs) that mediate stem cell activation, genome stability, and sustained survival, or death, in somatic stem cells (Gordon et al., 2018). On the one hand, the mitogenic Cyclin G1/Cdk (Cdk2 or Cdk5)/Myc/Pin1 Axis of PPIs govern early stem cell activation and the biochemical *Competence* to proliferate, serving as a sustained and sustaining *Survival Factor*; while genomic stability and DNA-repair mechanisms are governed largely by the Cyclin G1/PP2A/MDM2/p53/p18-Hamlet Axis of interacting proteins, which ensure DNA fidelity at defined cell cycle checkpoints, in coordination with the p53 tumor suppressor (Vousden and Carol Prives, 2009) and with p18-Hamlet, a Cyclin G1-binding phosphoprotein which serves as a molecular sensor of genomic stress within the molecular checks and balances that determine cell fate. (Cuadrado et al., 2007; Lafarga et al., 2007).

The epic translation of gene-based research to applied clinical oncology was facilitated by the development of a broadly cytocidal *dominant-negative* construct of the Cyclin G1 oncogene (dnG1), along with the molecular engineering of synthetic lesion-targeted gene therapy expression vectors guided by pathotropic targeting (Hall et al., 2010); i.e., by affinity for the exposed anaplastic collagenous extracellular matrix proteins that are characteristic signatures of the tumor microenvironment (Su and Karin, 2023). A testament to perseverance and quantitative analytical investigations in clinical medicine, the first reports of long-term (>10 years) cancer-free survival in patients with advanced, previously intractable, chemo-resistant, metastatic cancers of the pancreas, bone, and breast were formally published in 2021 (Liu et al., 2021), which amounts to somewhat more than an academic milestone. Indeed, the topical papers presented herein by Chawla et al. and Bruckner et al., describe the recent clinical revival, restored cGMP production, the latest regulatory approvals, and current clinical trials of DeltaRex-G for the treatment of advanced pancreatic cancer and sarcoma, and carcinoma of breast, respectively. Finally, the topical article presented by Ticha et al., explores the limits and potential role of minimally invasive localized venous/tumor DNA sampling methodologies to improve the sensitivity and utility of liquid biopsies to monitor biological markers, disease progression, and treatment responses in complex clinical settings.

In summary, the field of genetics has experienced momentous growth from Mendel’s pea plant experiments to the advent of an increasing number of gene therapy products for cancer and genetic disorders.
